# The Impact of Deoxynivalenol on Pigeon Health: Occurrence in Feed, Toxicokinetics and Interaction with Salmonellosis

**DOI:** 10.1371/journal.pone.0168205

**Published:** 2016-12-20

**Authors:** Gunther Antonissen, Roel Haesendonck, Mathias Devreese, Nathan Broekaert, Elin Verbrugghe, Sarah De Saeger, Kris Audenaert, Freddy Haesebrouck, Frank Pasmans, Richard Ducatelle, Siska Croubels, An Martel

**Affiliations:** 1 Department of Pharmacology, Toxicology and Biochemistry, Faculty of Veterinary Medicine, Ghent University, Merelbeke, Belgium; 2 Department of Pathology, Bacteriology and Avian Diseases, Faculty of Veterinary Medicine, Ghent University, Merelbeke, Belgium; 3 Department of Bioanalysis, Faculty of Pharmaceutical Sciences, Ghent University, Ghent, Belgium; 4 Department of Applied Biosciences, Faculty of Bioscience Engineering, Ghent University, Ghent, Belgium; University of Minnesota, UNITED STATES

## Abstract

Seed-based pigeon diets could be expected to result in exposure of pigeons to mycotoxins such as deoxynivalenol (DON). Ingestion of low to moderate contamination levels of DON may impair intestinal health, immune function and/or pathogen fitness, resulting in altered host-pathogen interactions and thus different outcome of infections. Here we demonstrate that DON was one of the most frequently detected mycotoxins in seed-based racing pigeons feed, contaminating 5 out of 10 samples (range 177–1,466 μg/kg). Subsequently, a toxicokinetic analysis revealed a low absolute oral bioavailability (F) of DON in pigeons (30.4%), which is comparable to other avian species. Furthermore, semi-quantitative analysis using high-resolution mass spectrometry revealed that DON-3α-sulphate is the major metabolite of DON in pigeons after intravenous as well as oral administration. Following ingestion of DON contaminated feed, the intestinal epithelial cells are exposed to significant DON concentrations which eventually may affect intestinal translocation and colonization of bacteria. Feeding pigeons a DON contaminated diet resulted in an increased percentage of pigeons shedding *Salmonella* compared to birds fed control diet, 87 ± 17% versus 74 ± 13%, respectively. However, no impact of DON was observed on the *Salmonella* induced disease signs, organ lesions, faecal and organ *Salmonella* counts. The presented risk assessment indicates that pigeons are frequently exposed to mycotoxins such as DON, which can affect the outcome of a *Salmonella* infection. The increasing number of pigeons shedding *Salmonella* suggests that DON can promote the spread of the bacterium within pigeon populations.

## Introduction

*Fusarium* is one of the most important toxigenic fungal genera in small grain cereals. Although members of this genus can cause grain yield losses, the interest in *Fusarium* head blight is primarily fuelled by the ability of the majority of the *Fusarium* species to produce mycotoxins. These mycotoxins exhibit an intrinsic toxicity and pose a potential acute or chronic health threat to humans and animals upon ingestion of contaminated food or feed [1, 2]. The mycotoxin deoxynivalenol (DON) is one of the most frequently detected mycotoxins in feed and feed raw materials in temperate climates as present in Western Europe [3]. DON is a type B trichothecene mainly produced by *F*. *graminearum* and *F*. *culmorum* [4]. DON has major negative effects on animal health and animal production. Birds, with chickens and turkeys as most intensively studied, are considered rather resistant to DON compared to monogastric mammals [5]. Differential sensitivity might be related to differences in toxicokinetic properties (absorption, distribution, metabolism and excretion) among animal species [5, 6]. After a single bolus administration, a low absolute oral bioavailability (F) of 19% and 21% was observed in fasted broiler chickens and turkeys, respectively [7, 8]. This is in contrast to pigs, which is one of most sensitive animal species for DON toxicity, where a complete oral absorption following single oral bolus administration was observed (F = 100%) [9]. Furthermore, compared to pigs a higher plasma clearance of DON was observed in broilers after intravenous administration [8]. This can be attributed to phase II biotransformation which is much more extensive in broilers than in pigs. DON biotransformation in pigs consists mainly of glucuronidation, and in chickens extensive sulfation predominantly occurs [7]. However, data describing these toxicokinetic characteristics in pigeons are lacking.

It is well known that DON acts as an inhibitor of the protein synthesis at the ribosomal level [10, 11]. Therefore, it is suggested that rapidly proliferating cells and tissues with high protein turnover rates, such as the immune system, liver, and gastrointestinal tract are most affected [12, 13]. DON negatively affects the intestinal barrier in many species by its impact on cell viability and proliferation, intestinal morphology, intestinal mucus layer, production of antimicrobial peptides, epithelial integrity, and modulation of digestive and absorptive processes [14–17]. Hence, this can lead to an enhanced susceptibility to enteric diseases, such as necrotic enteritis in broiler chickens [15, 18].

Since the intestinal tract is also a major portal of entry to many pathogens, DON exposure could affect the animal susceptibility to infectious diseases, such as salmonellosis [19, 20]. Feeding pigs a DON-contaminated diet may influence the intestinal phase of a *Salmonella* Typhimurium infection by enhancing *Salmonella* invasion and translocation across the intestinal epithelium, as well as by potentiating the early intestinal immune response induced by the infection [19, 20]. Furthermore, DON also altered the systemic phase of infection by promoting the uptake of *Salmonella* Typhimurium by macrophages, through modulation of the cytoskeleton due to extracellular signal-regulated kinase 1/2 (ERK1/2) activation [19]. In broiler chickens and mice challenged with another trichothecene mycotoxin T-2 toxin, an increased level of *Salmonella* Typhimurium-related organ lesions or mortality was seen [21–24]. Salmonellosis is the main bacterial disease in pigeons and typically associated with pigeon-adapted *Salmonella* Typhimurium variant Copenhagen strains [25, 26]. The disease is characterized by a variety of symptoms such as gastroenteritis, arthritis, oophoritis or orchitis, systemic granulomatous inflammation, and mortality [27].

The consumption of seed-based diets could result in high exposure to mycotoxins such as DON in pigeons, which in turn may affect the animal's susceptibility to infectious diseases [1]. Therefore, the aims were (1) to determine the occurrence of mycotoxins in commercial racing pigeon (*Columba livia*) feed, (2) to assess the toxicokinetic characteristics of DON in racing pigeons, and (3) to evaluate the impact of DON on the susceptibility and course of an experimental *Salmonella* Typhimurium variant Copenhagen infection in racing pigeons.

## Materials and Methods

### Mycotoxin occurrence in racing pigeon feed

Ten different commercial racing pigeon feeds, all seed mixtures, were bought from local stores in Flanders, Belgium. On average, the major commodities of these seed mixtures were corn (34 ± 6.9%), peas (17 ± 8.0%), wheat (15 ± 6.9%), sorghum (15 ± 8.4%), rice (7 ± 6.6%), barley (6 ± 3.8%) and safflower seed (6 ± 4.0%). Minor commodities in some of the seed mixtures were oats (3 ± 2.4%), hemp seed (2 ± 2%), sunflower seed (2 ± 0.6%), buckwheat (2 ± 1.4%), rape seed (2 ± 1.8%), linseed (1 ± 0.9%), milk thistle seed (1 ± 1%), millet (1 ± 0.4%) and canary seed (1 ± 0.5%). Samples of 500 g were taken at three different locations in one bag of each of the 10 feeds (20 kg), subsequently pooled and analyzed for mycotoxin contamination. Analysis was performed by a validated multi-mycotoxin liquid chromatography-tandem mass spectrometry method (LC-MS/MS) [28]. The following mycotoxins were analyzed: aflatoxin B_1_, B_2_, G_1_ and G_2_, altenuene, alternariol (AOH), alternariol methylether (AME), DON, 3-acetyl-deoxynivalenol (3ADON), 15-acetyl-deoxynivelenol (15ADON), diacetoxyscirpenol, enniatin B (ENNB), fumonisin B_1_ (FB_1_), B_2_ (FB_2_) and B_3_ (FB_3_), fusarenon-X, neosolaniol, nivalenol, ochratoxin A (OTA), roquefortine-C, sterigmatocystin, T-2 toxin, HT-2 toxin and zearalenone (ZEN). The decision limit (CCα) was defined as the concentration at “the y intercept plus 2.33 times the standard deviation of the within laboratory reproducibility” (α = 1%) in the case of substances for which no permitted limit has been established. In the case of substances with a maximum limit such as aflatoxin B_1_, CCα was established by spiking blank material around the maximum limit. The corresponding concentration at the permitted limit plus 1.64 times the standard deviation of the within laboratory reproducibility equals the decision limit (α = 5%) [28].

### Toxicokinetics of DON

#### Animal trial

Ten racing pigeons (5♂/5♀, 4 months old, body weight (BW): 430 ± 34 g) were obtained from a commercial breeder. A one week acclimatization period was respected and animals were group housed applying a 14 h light/10 h dark cycle. A seed-based diet (Table 1, feed n° 1) and water were given *ad libitum*. Animals were deprived of feed 8h before the administration of DON until 2 h post-administration (p.a.). Pigeons were administered a single bolus of DON (0.3 mg/kg BW), either by oral gavage (PO) (birds 1–5, 3♂/2♀) or by intravenous injection (IV) in the *vena cutanea ulnaris superficialis* (wing vein) (birds 6–10, 2♂/3♀), using a two-way crossover design. This administered dosage was based on the average daily feed intake (30 g per pigeon) and maximum European guidance level of DON in poultry feed (5,000 μg/kg feed). The DON bolus was prepared immediately prior administration by dissolving DON analytical standard (Fermentek, Jerusalem, Israel) in ethanol (10 mg/mL) and further diluting with water (PO bolus) or saline solution (IV bolus) up to a concentration of 0.3 mg/mL. Blood samples (0.2 mL) were collected in heparinized tubes from the *vena metatarsalis plantaris superficialis* (leg vein) before administration (t = 0) and at 5, 10, 20, 30, 40, 50, 60, 90 and 120 min p.a. Blood samples were centrifuged (2851 x g, 10 min, 4°C) and plasma was stored at ≤-15°C until further analysis. After a two weeks wash-out and recovery period, the protocol was repeated. The birds that previously received an IV injection of the mycotoxin then received a PO bolus and *vice versa*. The dosing, blood collection, and sample storage was performed in the same way as the first administration. The animal experiment was approved by the Ethical Committee of the Faculty of Veterinary Medicine and the Faculty of Bioscience Engineering of Ghent University (EC 2014/67).

**Table 1:** Occurrence of mycotoxins (μg/kg) in ten commercially available racing pigeon feed samples.

	pigeon feed									
	1	2	3	4	5	6	7	8	9	10
mycotoxin^1^										
**DON**	ND	340 ± 99	ND	230 ± 67	ND	177 ± 52	1,466±428	ND	ND	359 ± 105
**3-ADON**	6 ± 2	39 ± 15	ND	7 ± 3	ND	ND	13 ± 5	ND	ND	9 ± 4
**ZEN**	ND	76 ± 22	ND	ND	ND	54 ± 16	51 ± 15	ND	ND	ND
**AOH**	ND	85 ± 34	20 ± 8	25 ± 10	53 ± 21	27 ± 11	17 ± 7	99 ± 39	168 ± 67	81 ± 32
**AME**	ND	49 ± 19	ND	ND	ND	ND	ND	81 ± 31	172 ± 67	105 ± 41
**FB**_**1**_	ND	ND	1,070 ± 126	ND	ND	ND	ND	ND	ND	ND
**FB**_**2**_	ND	ND	181 ± 15	ND	ND	ND	ND	ND	ND	ND
**FB**_**3**_	ND	ND	169 ± 61	ND	ND	ND	ND	ND	ND	ND
**OTA**	ND	ND	16 ± 2	ND	ND	ND	ND	ND	ND	ND
**ENNB**^2^	<80μg/kg	<80μg/kg	<80μg/kg	<80μg/kg	<80μg/kg	<80μg/kg	>80μg/kg	<80μg/kg	>80μg/kg	<80μg/kg

^1^DON = deoxynivalenol; 3-ADON = 3-acetyldeoxynivalenol; AOH = alternariol; ZEN = zearalenone; AME = alternariol methylether; FB_1_ = fumonisin B_1_; FB_2_ = fumonisin B_2_; FB_3_ = fumonisin B_3_; OTA = ochratoxin A; ENNB = enniatin B

^2^ENNB is measured qualitatively. Mycotoxin concentrations are presented ± expanded measurement uncertainty. ND = not detected or below decision limit (CCα)

No levels above the CCα were detected of the following mycotoxins: aflatoxin B_1_, B_2_, G_1_ and G_2_, altenuene, 15-acetyldeoxynivalenol, diacetoxyscirpenol, fusarenon-X, neosolaniol, nivalenol, roquefortine-C, sterigmatocystin, T-2 toxin, and HT-2 toxin.

#### Analysis of DON and its metabolites in plasma and toxicokinetic analysis

**Fig 1:**
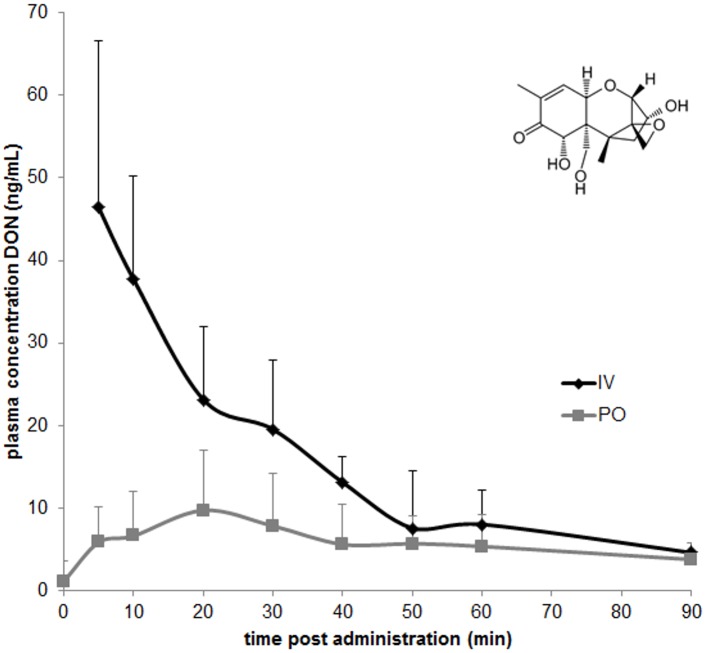
Chemical structure (insert) and plasma concentration-time profile of deoxynivalenol (DON) after single IV (n = 10) and PO (n = 10) administration of 0.3 mg DON/kg bodyweight to racing pigeons. Values are presented as means + SD.

DON analytical standard was purchased from Fermentek and was dissolved in acetonitrile (ACN) yielding a stock solution of 1 mg/mL (chemical structure in Fig 1). Deepoxydeoxynivalenol (DOM-1) (50 μg/mL) and ^13^C_15_-DON (stable isotope labelled internal standard (IS), 25 μg/mL) stock solution in ACN were purchased from Sigma-Aldrich (Diegem, Belgium). All the above mentioned stock solutions were stored at ≤−15°C. Individual working standard solutions of 1 μg/mL were prepared by diluting the above stock solutions with HPLC-grade ACN and were stored at ≤−15°C. ACN solutions of DON have been reported stable for 24 months at room temperature, for DOM-1 no published stability data are available [29]. However, the supplier claims a shelf-life of at least 6 months at 4°C for a 100 ng/mL solution in ACN.

Quantification of DON and DOM-1 in plasma was based on a validated LC-MS/MS method for broiler chicken plasma using matrix-matched calibration curves [30]. Only minor modifications were made with respect to the lower volumes of plasma. Therefore, the validation was limited to a re-assessment of the limit of detection (LOD) and limit of quantification (LOQ). LOQ was calculated as the lowest concentration for which the method had acceptable results with regards to accuracy and precision. It was determined by spiking 6 plasma samples at 0.1, 0.5, 1 or 2 ng/mL and it was set at 0.5 ng/mL for DON and 1 ng/mL for DOM-1. The LOQ was also established as the lowest point of the calibration curve. The LOD was calculated using the samples spiked at the LOQ level (n = 6) corresponding to the lowest concentration that could be determined with a signal-to-noise (S/N) ratio of 3.

Toxicokinetic analysis was performed using a non-compartmental model (WinNonlin 6.3, Pharsight, USA). Plasma levels below LOQ were not included in the toxicokinetic analysis. The most important toxicokinetic parameters of DON were calculated: maximal plasma concentration (C_max_), time to maximal plasma concentration (T_max_), area under the plasma concentration-time curve from time 0 to 90 min (AUC_0-t_), elimination rate constant (k_el_), elimination half-life (T_1/2el_), mean residence time (MRT), volume of distribution (Vd), and clearance (Cl). The absolute oral bioavailability (F) was calculated according to the following formula:

$$F\left(\%\right)=\frac{{\text{AUC}}_{0-\text{t PO}}}{{\text{AUC}}_{0-\text{t IV}}}\cdot 100$$

The samples of five pigeons, of both IV and PO administration of DON, were also analysed by (U)HPLC coupled to high resolution mass spectrometry (HR-MS) analysis for identification and semi-quantification of the phase II biotransformation metabolites of DON. Analysis was based on a method for turkeys and broiler chickens, as previously described by Devreese *et al*. [7].

### The impact of DON on the susceptibility and course of a *Salmonella* infection

#### Production of DON contaminated feed

DON contaminated feed was produced by adding DON to a control diet, a commercial extruded pellet feed (Versele Laga, Deinze, Belgium). DON was kindly provided by dr. M. Lemmens (BOKU, Vienna, Austria). Purity of the provided stock was > 99%. DON was dissolved in ethanol and further diluted in a fructose-glucose syrup (Isosweet, Tereos syral, Aalst, Belgium). Subsequently, this syrup was sprayed homogeneously over the pellets to a final level of 2% (v/m). Similarly, a blank syrup was sprayed over the control diet. Mycotoxin contamination of both the control and DON contaminated diet was analyzed by the validated multi-mycotoxin LC-MS/MS method as mentioned in section 2.1 [28]. Low levels of 15-ADON (44 ± 22 μg/kg) and ZEN (113 ± 33 μg/kg), and trace amounts of nivalenol (40 ± 12 μg/kg), DON (218 ± 64 μg/kg), 3-ADON (9.0 ± 3 μg/kg), FB_1_ (42 ± 5 μg/kg) and FB_2_ (80 ± 8 μg/kg) were detected in the control feed. The DON level in the mycotoxin contaminated diet was 3,538 ± 1,000 μg/kg feed.

#### *In vivo* infection trial

Twenty racing pigeons were divided into two groups (5♂/5♀, 4 months old), one group was fed the control diet and the other group was fed the DON contaminated diet. The animals were obtained from a breeding colony free of *Salmonella* and were negative for the presence of *Salmonella* bacteria in their feces at multiple sampling points as well as for serum agglutinating antibodies and the presence of anti-*Salmonella* IgG antibodies as tested by an in-house enzyme-linked immuno-sorbent assay (ELISA). Feed and water were given *ad libitum* throughout the trial and a 14 h light/ 10 h dark cycle was applied. During a two week acclimatization period all animals were fed the control diet and group housed in an aviary of 6 m^3^.

Next, experimental diets, i.e. control or DON contaminated feed, were fed from day 1 of the experiment onwards until the end of the experiment (day 28). From day 1 until day 7 birds were housed per experimental group in an aviary. From day 8 onwards each animal was housed individually in a cage of 50 x 70 x 40 cm (width x depth x height) with a wire mesh floor. The BW of animals was measured on day 7, 14, 21 and 28. A pigeon-adapted *Salmonella* Typhimurium variant Copenhagen DAB69 strain [25] was grown overnight in Luria-Bertani broth (LB) at 37°C, and at day 15, each pigeon was inoculated in the crop with 1 mL of inoculum containing 10^8^ colony forming units (CFU) of the strain. Post inoculation, *Salmonella* fecal shedding was assessed daily by counting the numbers of *Salmonella* CFU per gram excreta. This was done by plating ten-fold serial dilutions on Brilliant Green Agar (BGA) plates (LabM, Lancashire, UK). If negative after direct plating, the samples were pre-enriched in buffered peptone water (Oxoid, Basingstoke, UK) at 37°C and then enriched in tetrathionate brilliant green broth (Merck KGaA, Darmstadt, Germany) at 37°C. The animals were examined daily for the presence of disease signs such as apathy, diarrhea, and polyuria. Faecal consistency was scored daily as described by Pasmans *et al*. [25]: 0, normal faeces; 1, faeces not well formed; 2, watery faeces; 3, severe diarrhea; 4, hematochezia; 5, absence of faecal production combined with anorexia. At day 28 (13 days post inoculation) all pigeons were euthanized by an IV injection of sodium pentobarbital (0.5mL/kg; Natrium Pentobarbital 20%, Kela Veterinaria, Sint-Niklaas, Belgium) and necropsied. Complete lung, liver, spleen, kidney, gonads, brain, and joints were scored exclusionary (and measured in case of granulomas)–with the highest score being chosen–for the presence of *Salmonella* lesions by the same person as follow: 0, no macroscopic lesions; 1, visual organ enlargement; 2, presence of granuloma’s (< 3 mm); 3, presence of granuloma’s (3–6 mm); 4, presence of granuloma’s (7–9 mm); 5, presence of granuloma‘s (> 9 mm). The small intestines were scored exclusionary using the following system: 0, no macroscopic lesions; 1, serosal congestion; 2, abnormal content; 3, haemorrhagic content; 4, presence of granuloma’s (<3 mm); 5, presence of granuloma‘s (3–6 mm). From each animal, the left lung, liver, spleen, kidney, gonads, brain, ileal mucosa, ileal content were homogenized, and the number of CFU of *Salmonella* per g tissue was determined as described above. Tissue samples were fixed in 10% buffered formalin. After fixation, the samples were processed for histological examination. Paraffin sections were stained with haematoxylin-eosin. Ten serial histologic sections of three randomly selected lung samples per group were submitted to further histologic examination. The animal experiment was approved by the Ethical Committee of the Faculty of Veterinary Medicine and Bioscience Engineering of Ghent University (EC 2014/108).

#### *In vitro* evaluation of the effect of DON on growth of *Salmonella* Typhimurium variant Copenhagen strain DAB69

Following concentrations of DON were tested for their effect on *Salmonella* growth: 0, 0.2, 2 and 20 μg DON/mL. A stationary phase culture of *Salmonella* Typhimurium variant Copenhagen strain DAB69 was 1:2500 diluted in LB broth medium, and incubated aerobically at 37°C. Tenfold dilutions were prepared in phosphate buffered saline (PBS), and subsequently plated on BGA at 0, 1, 2, 3, 4, 5, 6, 7, 8, and 24h after incubation to determine the number of CFU/mL and establish a growth curve. After aerobic incubation overnight at 37°C, the number of CFU per mL was determined. All tests were performed in triplicate.

### Statistics

Statistical analysis was done using SPSS 23 software (IBM, USA). BW and number of *Salmonella* CFU were tested for normality using the Shapiro-Wilk test. Normally distributed data were analyzed using the (un)paired two-sided Student’s t-test to address the significance of difference between mean values with significance set at p ≤ 0.05. Categorical dependent variables faecal consistency score and *Salmonella* lesion score were converted to a binary dependent variables, with a score of two or more considered as poor faecal consistency and positive for *Salmonella*-related organ lesions, respectively. Subsequently, faecal consistency, *Salmonella* lesion score, and percentage of pigeons shedding *Salmonella* per day were analyzed by logistic regression.

## Results

### Mycotoxin occurrence in pigeon feed

DON was detected in 5 out of 10 commercial pigeon feed samples (CCα = 61 μg/kg), with an average contamination level of 515 ± 537 μg/kg (range 177–1,466) (Table 1). However, even the highest concentration of DON detected in this study (1,466 μg/kg), was in accordance with the European maximum guidance level of DON in poultry feed (5,000 μg/kg) [31]. Besides DON, most pigeon feed samples were also contaminated with following mycotoxins: AOH (9 out of 10 samples, 64 ± 50 μg/kg) (CCα = 12 μg/kg), 3-ADON (5 out of 10, 15 ± 14 μg/kg) (CCα = 5 μg/kg), AME (4 out of 10, 102 ± 52 μg/kg) (CCα = 18 μg/kg) and ZEN (3 out of 10, 60 ± 14 μg/kg) (CCα = 18 μg/kg) (Table 1). One feed sample was also contaminated with fumonisins (1,070 μg FB_1_/kg, 181 μg FB_2_/kg, and 169 μg FB_3_/kg) (CCα FB_1_ = 32 μg/kg, FB_2_ = 24 μg/kg and FB_3_ = 23 μg/kg) and OTA (16 ± 1.9 μg/kg) (CCα = 3 μg/kg). None of the observed contamination levels of ZEN, fumonisins, and OTA exceeded the European maximum guidance level for poultry feed [31]. Currently, no guidance levels are defined for acetylated DON and the *Alternaria* mycotoxins AOH and AME. No levels above the CCα were detected of the following mycotoxins: aflatoxin B_1_ (CCα = 7 μg/kg), B_2_ (CCα = 2 μg/kg), G_1_ (CCα = 2 μg/kg) and G_2_ (CCα = 2 μg/kg), altenuene (CCα = 5 μg/kg), 15ADON (CCα = 3 μg/kg), diacetoxyscirpenol (CCα = 1 μg/kg), fusarenon-X (CCα = 17 μg/kg), neosolaniol (CCα = 7 μg/kg), nivalenol (CCα = 36 μg/kg), roquefortine-C (CCα = 1 μg/kg), sterigmatocystin (CCα = 5 μg/kg), T-2 toxin (CCα = 9 μg/kg), and HT-2 toxin (CCα = 9 μg/kg).

### Toxicokinetics of DON

**Fig 2:**
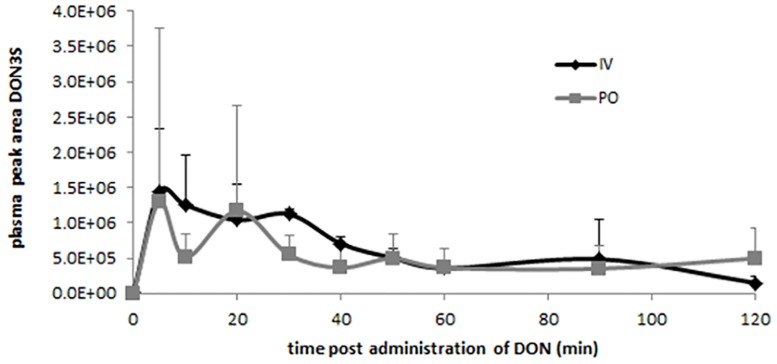
Peak area of deoxynivalenol-3alpha-sulphate (DON3S) at different time points after intravenous (IV) (n = 5) and oral (PO) (n = 5) administration of 0.3 mg DON/kg body weight to pigeons, as determined by high resolution-mass spectrometry. Values are presented as means + SD.

Toxicokinetic analysis was performed using a non-compartmental model, an acceptable curve fitting could be achieved for 10 pigeons after IV administration, but only for 6 pigeons after PO administration. The mean plasma concentration-time profile and the main toxicokinetic characteristics after IV and PO administration of DON are presented in Fig 1 and Table 2, respectively. As can be observed, the absolute oral bioavailability (F) of DON following PO administration was 30.4%. The Vd and Cl following PO administration were corrected for the absolute oral bioavailability. DON was rapidly eliminated after IV (T_1/2el_ = 0.3 ± 0.09 h), as well as after PO (T_1/2el_ = 0.6 ± 0.19 h) administration. DOM-1 was not detected in any sample. The HR-MS analysis revealed the presence of the deoxynivalenol-3a-sulphate (DON3S) metabolite in plasma (Fig 2). DOM-1, DON-3α-glucuronide, 10-DON-sulphonate, 10-de-epoxydeoxnivalenol-sulphonate were not detected in any sample by HR-MS analysis.

**Table 2:** Main toxicokinetic parameters (mean ± SD) of DON after single IV (n = 10) and PO (n = 6) bolus administration of 0.3 mg/kg BW to racing pigeons.

	IV	PO
AUC_0-90_ (ng*h/mL)	27.0 ± 8.4	8.2 ± 4.5
C_max_ (ng/mL)	-	10.1 ± 5.2
C_0_ (ng/mL)	94.9 ± 45.4	-
Tmax (h)	-	0.5 ± 0.2
k_el_ (/h)	2.3 ± 0.7	1.2 ± 0.4
t_1/2el_ (h)	0.3 ± 0.1	0.6 ± 0.2
Vd ^a^(L/kg)	5.7 ± 18	28.1 ± 15.2
Cl ^b^ (L/h*kg)	12.5 ± 4.8	31.8 ± 15.2
F(%)	100	30.4

AUC_0-90_, area under the curve from time 0 to 90 minutes post administration; C_max_, maximum plasma concentration; C_0_, plasma concentration at time = 0; T_max_, time of maximum plasma concentration; k_el_, elimination rate constant; t_1/2el_, half-life of elimination; Vd, volume of distribution; Cl, clearance; F, absolute oral bioavailability. The Vd ^(a)^ and Cl ^(b)^ of DON following PO administration were corrected for the absolute oral bioavailability.

### Impact of DON on the susceptibility and course of a *Salmonella* infection

**Fig 3:**
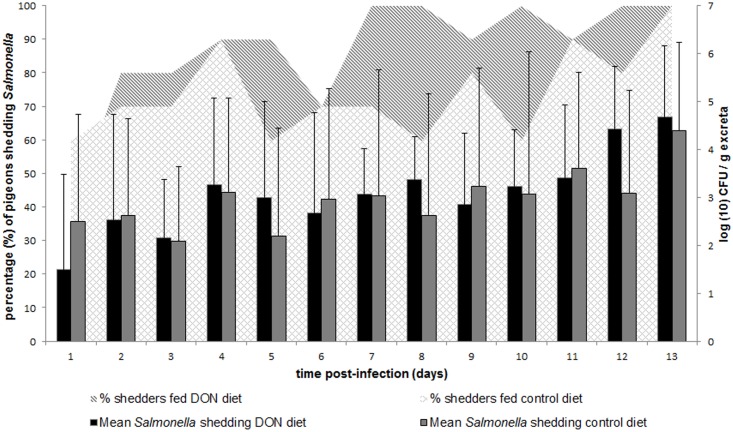
*Salmonella* fecal shedding. Pigeons were fed either a control diet or a DON contaminated diet for 28 days. Post inoculation with a pigeon-adapted *Salmonella* Typhimurium variant Copenhagen strain DAB69, *Salmonella* fecal shedding was assessed daily by counting the numbers of *Salmonella* in excreta samples. Results are presented as the mean number (+ SD) of CFU of *Salmonella* per gram of excreta and the percentage of pigeons shedding *Salmonella* per experimental group.

**Fig 4:**
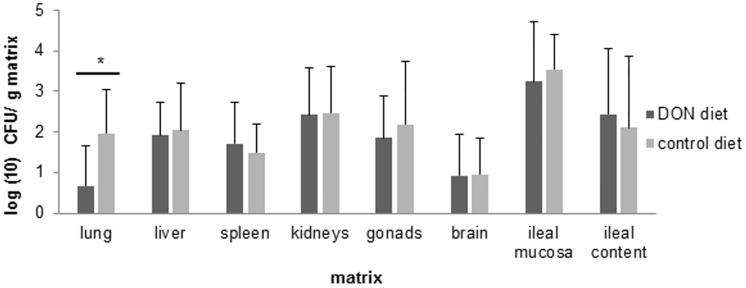
*Salmonella* numbers in organs. Thirteen days post inoculation with *Salmonella* Typhimurium variant Copenhagen strain DAB69, birds were euthanized and organs were collected, homogenized, and examined for the number of CFU of *Salmonella* per gram of matrix. Results are presented as mean + SD.

Feeding pigeons a diet contaminated with DON significantly increased the percentage of pigeons shedding *Salmonella* per day compared to the control group, 87 ± 17% versus 74 ± 13% (*P = 0*.*04*), respectively (Fig 3). However, no difference between the experimental groups was observed in the mean number of *Salmonella* CFU in the excreta samples (Fig 3). No significant difference in the percentage of birds with poor fecal consistency per day was observed between pigeons fed a DON contaminated diet and pigeons fed a control diet, 19 ± 18% versus 24 ± 23%, respectively (*P = 0*.*71*). Anorexia was only observed in one bird in the DON group and two birds in the control group. Polyuria was only observed in two pigeons in the DON group and not in the control group. Following the oral inoculation with *Salmonella*, a decrease in the bird’s BW was observed, although no difference was observed between both experimental groups (*P> 0*.*1)* (S1 Table). None of the pigeons in both groups were positive (organ lesion score ≥ 2) for the presence of *Salmonella*-related lesions in lung, gonads, brain and joints. However, a large percentage of pigeons showed *Salmonella*-related lesions in liver (90%–90%), kidneys (60%–70%) and small intestines (50%–60%), in both, control and DON group, respectively. In only one animal in the control group and none in the DON group a *Salmonella*-related lesion was observed in the spleen. No significant differences were observed between both experimental groups. In contrast, a significant lower mean number of *Salmonella* CFU (*P = 0*.*01*) was observed in the lung of pigeons fed a DON contaminated diet, compared to pigeons fed a control diet (Fig 4). *Salmonella* could also be detected in the lung of a lower percentage of birds in the DON group (40%) compared to the control group (90%), and only in one pigeon in the control group and none in the DON group organ lesions were observed. Histology of the lung revealed typical lesions seen in *Salmonella* infections. Within the DON group lymphocyte aggregates were found throughout the lung parenchyma and granuloma’s consisting of central necrosis surrounded by giant cells. The control group showed lymphocyte aggregates in the lung parenchyma, but in lower numbers and smaller in size compared to the ones in the DON group. Granuloma’s (histologically) were not present within the control group. The results of the *in vitro Salmonella* growth assay showed no influence of 0, 0.2, 2 or 20 μg DON/mL on the bacterial growth curve (S1 Fig).

## Discussion

For the first time, to our knowledge, a risk assessment was made for the mycotoxin DON in pigeons. The occurrence of different mycotoxins was clearly demonstrated in commercially available racing pigeons feed samples. The percentage of pigeon feed samples contaminated with DON and the average contamination level were in accordance with a long-term global mycotoxin survey program in feed and feed raw materials [32]. In this study, DON was detected in 5 out of 10 pigeon feed samples, with an average contamination level of 515 μg/kg. Similarly, the mycotoxin survey showed that 55% of 15,549 feed and feed raw material samples were contaminated with DON, with an average level of 535 μg/kg [32]. However, even the highest concentration of DON detected in this study (1,466 μg/kg), was in accordance with the European maximum guidance level of DON in poultry feed (5,000 μg/kg) [31].

It is important to emphasize the frequent co-occurrence of different mycotoxins in feed and feed raw materials. Indeed, different fungi are able to simultaneously produce a variety of mycotoxins, and on the other hand, any given commodity is likely to be infected with different types of fungi [33]. Moreover, since compound pigeon feed is made up of a number of different commodities, this will influence the final mycotoxin profile. The most important commodities of pigeon feed in this study were corn, peas, wheat and sorghum. Simultaneous presence of low doses of different mycotoxins in the diet may be more toxic than predicted from the mycotoxins alone [34, 35]. Besides the presence of DON and its acetylated forms, it was remarkable that 90% of the samples were also contaminated with *Alternaria* mycotoxins. However, the impact of *Alternaria* mycotoxins on animal health is still largely unknown [36, 37].

Differential sensitivity to the toxic effects of DON might be related to differences in toxicokinetic properties among animal species [5, 6]. The absolute oral bioavailability (F) of DON is slightly higher (30%) in fasted pigeons compared to fasted broiler chickens (19%) and turkeys (21%) [7, 8]. However, this low F suggests that intestinal epithelial cells are exposed to significant DON concentrations which eventually may affect intestinal translocation and colonization of bacteria. Furthermore, after IV injection of DON the mean volume of distribution (Vd) was similar in pigeons (5.7 L/kg) compared to broiler chickens (5.0 L/kg) and turkeys (7.4 L/kg), and total body clearance of DON was higher in pigeons (12.5 L/h*kg), compared to broiler chickens (7.2 L/h*kg) and turkeys (8.2 L/h*kg) [7, 8]. Consequently, this resulted in a shorter mean elimination half-life of DON in pigeons (0.34 h) compared to broilers (0.47 h) and turkeys (0.62 h). Next, it was demonstrated for the first time that DON is rapidly and extensively metabolised to DON3S. These results are in similarity with broiler chickens and turkey poults [7]. No DOM-1, DON-3α-glucuronide, 10-DON-sulphonate, 10-de-epoxydeoxnivalenol-sulphonate could be demonstrated in the plasma of pigeons. Also in broiler chickens and turkey poults it was demonstrated that glucuronidation of DON is a minor biotransformation pathway [7, 38]. Since, no standard solutions are commercially available for these metabolites (except for DOM-1) accurate quantitative analysis cannot be performed. Furthermore, the limited blood sampling in the toxicokinetic study of DON in pigeons could impair the detection of traces of different DON metabolites.

As salmonellosis is the main bacterial disease in pigeons [25, 26], it is important to know which factors are able to aggravate an infection. As DON is known to alter the intestinal barrier [14, 15, 17], an impact of DON on disease susceptibility could be assumed. However, the exact outcome of co-exposure to *Fusarium* mycotoxins and *Salmonella* infection is difficult to predict, and can be influenced by the animal host species, the bacterial strain and the effect of the mycotoxin on the bacterium, the host cells and the host-pathogen interaction. In the present study, it was demonstrated for the first time that feeding pigeons a diet contaminated with DON increased the percentage of pigeons shedding *Salmonella* compared to pigeons fed a control diet. This increasing number of pigeons shedding *Salmonella* might suggest that DON may promote the spread of the bacterium within or to other pigeon populations. As horizontal transmission of enteric pathogens is important in a population, the pathogen will benefit from an increasing number of individuals able to spread the pathogen. For pathogens which can induce a chronic carrier state and need a stable pathogen reservoir, a higher number of pathogen excreting animals is beneficial. A rapid and efficient intra- and inter-flock spread is primordial for horizontal transmission, especially in the case of pigeon salmonellosis. The difference in percentage of animals shedding the bacterium was not reflected in differences in the faecal and organ *Salmonella* counts between both groups in the present experimental setting. Besides, no impact of DON was observed on the occurrence of *Salmonella*-related clinical symptoms and organ lesions. Similarly, no obvious impact on *Salmonella* Typhimurium translocation could be demonstrated in pigs fed a fumonisins contaminated feed [39]. In contrast, feeding pigs a DON or T-2 toxin contaminated diet enhanced the intestinal *Salmonella* Typhimurium invasion and translocation [19, 40], although the study by Verbrugghe *et al*. [40] revealed lower cecal *Salmonella* counts correlated to the direct toxic effect of T-2 toxin on the bacterium. Although, no such direct toxic effect of DON was observed on the pigeon-adapted *Salmonella* Typhimurium variant Copenhagen strain DAB69, it remains unclear why the DON-exposed pigeons had lower lung *Salmonella* counts. Contradictorily, histologic examination of randomly chosen lungs showed more and more severe lesions in the DON group compared to the control group. It is important to emphasize that *Salmonella* organ count is not positively correlated with the number and severity of lesions. The fact that more microscopical lesions were noticed in the DON group might be related to the immunomodulating effect of DON [41].

In conclusion, our study clearly demonstrated that DON frequently contaminates feed for racing pigeons. Toxicokinetic analysis revealed that the absolute oral bioavailability of DON in pigeons was low, and comparable to other avian species. DON showed a high clearance, which results in a short elimination half-life. In accordance to other avian species, DON is predominantly metabolized to DON3S in pigeons. Feeding a DON contaminated diet may modulate the spread of a *Salmonella* infection in a pigeon flock by increasing the number of pigeons shedding the bacterium and a tendency towards more and extensive paratyphoid lesions. Future work looking at dose response, the effect of chronic exposure and the effect of DON in chronic *Salmonella* infections should be able to clarify the complex interplay between host, mycotoxin and pathogen.

## Supporting Information

S1 Table Bodyweight (BW) of pigeons in *in vivo Salmonella* infection trial. Pigeons were fed either a control diet or a DON contaminated diet for 28 days. BW was measured at day 7 (one day prior individual housing), day 14, 21 and 28. At day 15, each pigeon was inoculated with 10^8^ colony forming units of *Salmonella* Typhimurium variant Copenhagen DAB69 strain.
(TIFF)

S1 Fig *In vitro* evaluation of the effect of DON on growth of *Salmonella* Typhimurium variant Copenhagen strain DAB69. A stationary phase culture of *Salmonella* Typhimurium variant Copenhagen strain DAB69 was 1:2500 diluted in LB broth medium with 0, 0.2, 2 and 20 μg DON/mL, and incubated aerobically at 37°C. Samples were taken at 0, 1, 2, 3, 4, 5, 6, 7, 8 and 24h after incubation following inoculation with *Salmonella*. The number of colony forming units (cfu) per mL was determined by bacterial plating of 10-fold dilutions. Results are presented as the mean cfu/mL. There is no significant difference between the different test conditions.
(TIFF)
