# Supplementary figures and images for: The Impact of Deoxynivalenol on Pigeon Health: Occurrence in Feed, Toxicokinetics and Interaction with Salmonellosis
Source: PLoS One. 2016 Dec 20;11(12):e0168205. doi: 10.1371/journal.pone.0168205 (PMC5172580; doi:10.1371/journal.pone.0168205)

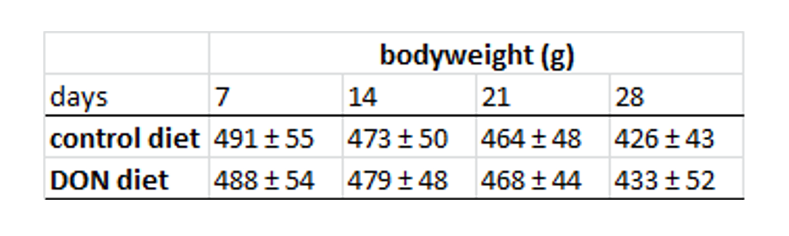

Supplement: S1 Table — Pigeons were fed either a control diet or a DON contaminated diet for 28 days. BW was measured at day 7 (one day prior individual housing), day 14, 21 and 28. At day 15, each pigeon was inoculated with 108 colony forming units of Salmonella Typhimurium variant Copenhagen DAB69 strain. (TIFF) [file pone.0168205.s001.tiff]

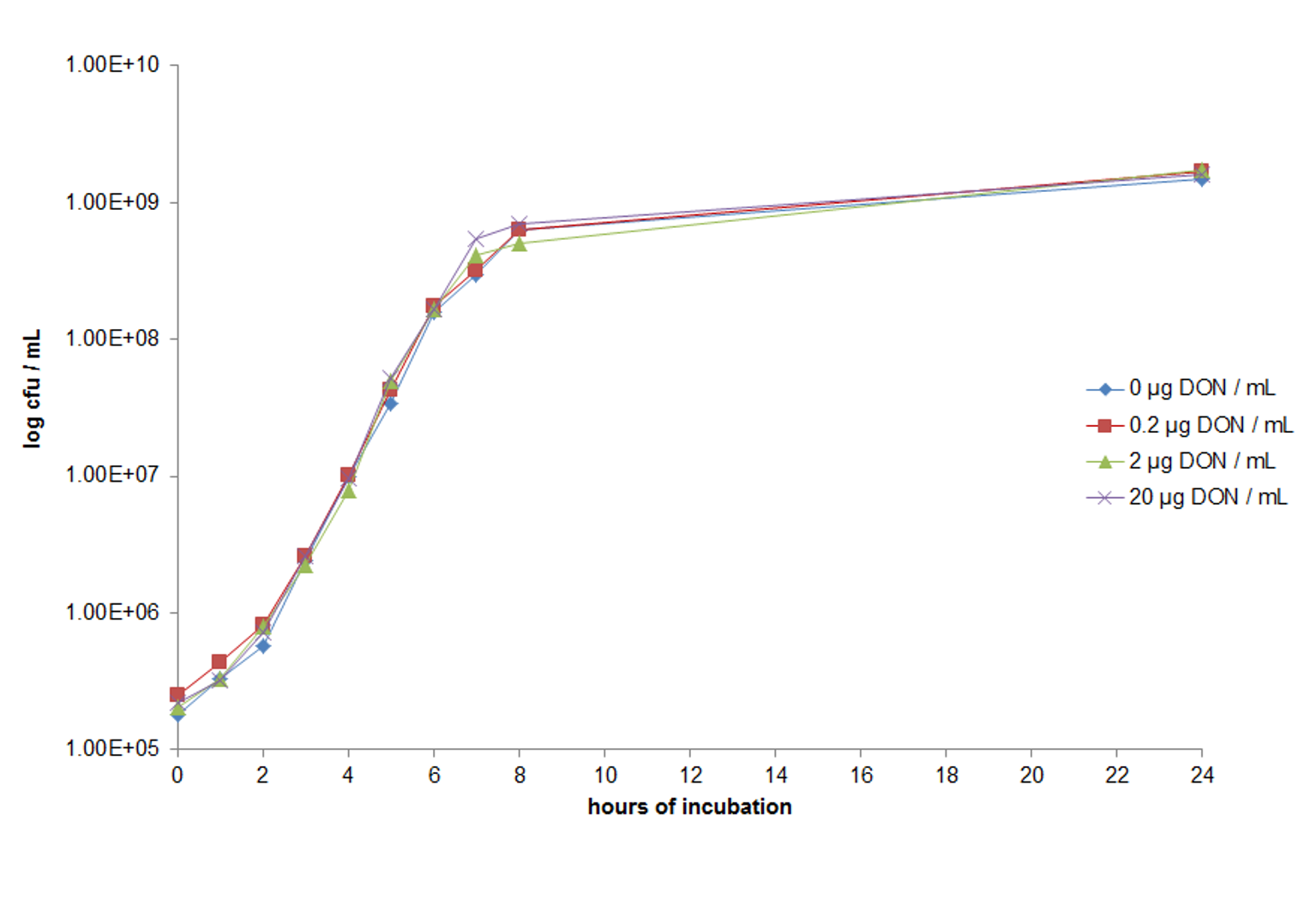

Supplement: S1 Fig — A stationary phase culture of Salmonella Typhimurium variant Copenhagen strain DAB69 was 1:2500 diluted in LB broth medium with 0, 0.2, 2 and 20 μg DON/mL, and incubated aerobically at 37°C. Samples were taken at 0, 1, 2, 3, 4, 5, 6, 7, 8 and 24h after incubation following inoculation with Salmonella. The number of colony forming units (cfu) per mL was determined by bacterial plating of 10-fold dilutions. Results are presented as the mean cfu/mL. There is no significant difference between the different test conditions. (TIFF) [file pone.0168205.s002.tiff]
